# Exploring the Integration of Trauma‐ and Violence‐Informed Research Principles in Longitudinal Youth Mental Health Research: An Arts‐Based Protocol

**DOI:** 10.1111/hex.70941

**Published:** 2026-10-29

**Authors:** Alexe Bernier, Zita de Snoo, Nawal Masood, Jawahir Al Bayati, Louise Gallagher, Melissa Kimber

**Affiliations:** ^1^ Offord Centre for Child Studies McMaster University Hamilton ON Canada; ^2^ McMaster Children's Hospital, Hamilton Health Sciences Hamilton ON Canada; ^3^ University of Toronto Toronto ON Canada; ^4^ The Hospital for Sick Children Toronto ON Canada; ^5^ Centre for Addiction and Mental Health Toronto ON Canada; ^6^ Department of Psychiatry and Behavioural Neurosciences McMaster University Hamilton ON Canada

## Abstract

**Background:**

Youths' engagement and participation experiences within quantitative, longitudinal mental health studies remain underexplored. Youth with complex mental health needs have unique research participation experiences, underscoring the need for research approaches that strengthen relationality, trust, and engagement. Trauma‐ and violence‐informed research (TVIR) principles offer a promising framework to address these challenges; however, youth‐centred perspectives on how TVIR is operationalized within longitudinal mental health research remains limited.

**Objective:**

CALM STORY will explore youths' perspectives and experiences of participating in longitudinal quantitative mental health research that is grounded in TVIR principles, with attention to choice, collaboration, connection, and control.

**Design:**

This protocol outlines CALM STORY, a qualitative, arts‐based longitudinal evaluation of youth participation in the Cohort Network of AdoLescents and youth with Multiple mental health conditions (CALM) Master Observational Trial (MOT). CALM STORY is theoretically organised around both TVIR principles as well as the Lundy Model of Participation.

**Methods:**

We are purposefully sampling 30 youth aged 11–19 years enroled in the CALM MOT. Youth are completing two semi‐structured qualitative interviews and a PhotoVoice workshop series. With consent, we will extract key socio‐demographic data from the CALM MOT to contextualise TVIR strategies. Data will be managed using NVivo software and thematically analysed using a framework‐based analytic approach guided by TVIR principles and the Lundy Model of participation.

**Conclusion:**

CALM STORY will generate youth‐centred insights into TVIR engagement and participation in longitudinal quantitative mental health research. Findings will inform key deliverables, including the development and scaling TVIR training for research teams, as well as a research policy brief to be disseminated to the CALM MOT network, research ethics boards, and research funders.

**Patient or Public Contribution:**

A Youth Advisory Group (YAG) comprised of youth with lived experience of multiple mental health conditions was established to consult on the CALM MOT's design, data collection, knowledge mobilisation, and sub‐studies. The YAG informed CALM STORY's study design, including the interview and PhotoVoice guides and will continued to be consulted throughout the study and its dissemination activities.

## 1 Introduction

Longitudinal studies have been instrumental in advancing knowledge of child and youth mental health. In Canada and beyond, mental health researchers have sought to learn more about how mental health conditions may develop and manifest over the course of young peoples' lives (e.g., [1, 2]). While this research has contributed important new knowledge to inform evolving evidence about how to better support youth with mental health challenges, little has been done to explore the experiences of the young people who engage with and participate in quantitative longitudinal mental health research processes or procedures.

To date, efforts around how best to engage and retain youth in longitudinal quantitative and qualitative research have focused on child and adolescent health research more generally [3, 4, 5]. Some key learnings about engaging youth in longitudinal health research have included the necessity of building strong relationships with youth participants and their caregivers [5, 6], incentivizing children and youth for their participation [4, 6], and conducting research that youth perceive as important and contributing to the greater social good [7]. While these insights are valuable, there are several facets of longitudinal mental health research with youth, specifically, that warrant focused attention.

For youth who are experiencing mental health challenges, the repetition of highly structured assessment or measurement processes may feel impersonal, invalidating or misaligned with their evolving self‐concept of how and who they are [8]. If youth have particularly complex lives, including but not limited to involvement with the child welfare system, housing precarity, or unstable caregiving arrangements, arranging for study follow up and participation can be complicated [5]. Additionally, adolescence and young adulthood is a period of significant identity formation: how youth understand, communicate, and share the various aspects of their identities can evolve rapidly [9, 10]. Contrastingly, quantitative measures and study designs often assume stability; this juxtaposition reveals itself by youth feeling misclassified or unseen by research that no longer aligns with who they are or the contextual influences on their well‐being [11], signalling a need for further investigation into how longitudinal study designs can better accommodate young people with diverse and complex needs and lives.

A potential approach for creating research environments that encourage and support sustained participation among youth experiencing mental health challenges is the integration of trauma‐ and violence‐informed care (TVIC) principles into research activities. Over the last decade, there has been a surge in appreciation for grounding health and mental health services within a TVIC approach (e.g., [12, 13, 14]). However, the consideration and articulation of trauma‐ and violence informed approaches in research settings is more recent and has been predominantly in qualitative research settings where the research focus is trauma or violence (e.g., [15, 16, 17]). There is limited information about the ‘how,’ ‘value,’ and ‘impacts’ of integrating TVIC principles into research more broadly and henceforth referred to as trauma‐ and violence‐informed research (TVIR). Critically, little evidence captures the value, impact, and experience of TVIR approaches, in any research setting, from youth's perspectives.

Integrating a TVIR approach into research with youth, regardless of research focus, is important because research constitutes a social and relational encounter that can either mitigate or exacerbate harm. Young people may enter research settings with diverse, and often undisclosed experiences of adversity, marginalisation, loss, or prior negative interactions with institutions, all of which can shape how they interpret questions, perceive safety, and engage with research staff and activities [18]. Aligned with TVIC, TVIR recognises these experiences as common and consequential, and that seemingly neutral research procedures (e.g., consent processes, standardised measures, repeated follow‐up, interactions with the research team, etc.), may evoke distress, mistrust, or disengagement [19, 20]. By attending to power dynamics, developmental stage, skill development, and the broader social context of young people's lives, TVIR aims to enhance ethical, equitable, safe, and sustained participation, thereby strengthening data quality, retention, and validity of longitudinal youth mental health research [18, 21, 22, 23].

### 1.1 Study Background

This study, titled “using the artS to Explore the Integration of Trauma‐ and ViOlence‐Informed Research Principles in Longitudinal Youth Mental Health Research (CALM STORY)”, will take place alongside the “Cohort Network of AdoLescents and youth with Multiple m ental health conditions Master Observational Trial” (i.e., CALM MOT). The CALM MOT is a Canadian multi‐site, longitudinal quantitative research study intending to advance understanding about the mental health trajectories of youth aged 11–24, with an emphasis on the mechanistic determinants of and interventions for multiple mental health conditions (MMHC) [24, 25]. Recruiting youth from six specialty mental health care sites, CALM MOT participants consent to completing: self‐report questionnaires about mental health symptoms, functioning, and context; structured diagnostic interviews capturing key clinical conditions; and providing core physiological and biological data. Data collection begins at baseline, with follow‐up at 3 months, and then at regular bi‐annual intervals, with some data collection elements repeated annually. The CALM MOT protocol is registered with ClinicalTrials. gov (NCT07699159) and detailed elsewhere [25].

TVIR approaches have been intentionally integrated into the CALM MOT [25]. Consistent with master observational trial designs [26, 27, 28, 29], which rely on harmonised core elements while permitting site‐level adaptation, the CALM MOT includes standardised TVIR elements across sites alongside context‐specific variation in how TVIR principles are operationalized. With respect to harmonisation, staff at all CALM MOT sites receive foundational training in TVIR based on a pan‐Canadian evidence‐based guidance [18]. Within this shared training framework, individual sites retain flexibility in how TVIR principles are embedded into local research procedures, workflows, and participant interactions. The McMaster Children's Hospital, Hamilton Health Sciences (MCH‐HHS) site is distinct from its sister sites, via an explicit operational emphasis on integrating TVIR principles into the research environment and experience. At MCH‐HHS, TVIR principles have been embedded within standard operating procedures (SOPs) and reinforced through ongoing training and reflective supervision [30], shaping how longitudinal research encounters are conducted. In practice, this has included: (a) continuity of research staff‐participant relationships, (b) structured “warm handoffs” during staffing transitions, (c) best practices in recognising and responding to suspected child maltreatment; (d) collaborative creation of a Crisis Response Plan with each research participant (detailed below), as well as (e) attention to systemic violence and participants' basic needs in protocol administration. This site‐level configuration provides a methodologically appropriate and theoretically informed context in which to examine how TVIR principles are experienced by youth participating in longitudinal quantitative mental health research.

### 1.2 Study Objectives

CALM STORY's primary objective is to understand how youth (11–19 years) enroled in the CALM MOT at MCH‐HHS perceive, interpret, and experience longitudinal quantitative research participation when TVIR principles are embedded in study design and procedures. Secondary objectives include exploration of their specific experiences of (a) creating and using a Crisis Response Plan as part of TVIR implementation for the CALM MOT at the MCH‐HHS site; and (b) opportunities for choice, connection, collaboration, and control over 1 year of participation in the CALM MOT. These objectives will be addressed through two semi‐structured interviews and a PhotoVoice workshop series that elicit youth's perspectives and experiences of TVIR in the CALM MOT more generally.

## 2 Methods

### 2.1 Study Design

We will achieve our research aims using a qualitative, multimethod, arts‐based longitudinal design. Specifically, a purposeful sample of youth enroled in the CALM MOT at MCH‐HHS will be approached to participate in CALM STORY research activities. For CALM STORY, youth will be asked for their informed consent to complete semi‐structured qualitative interviews, engage in doodling activities and PhotoVoice workshops, and permit secondary analysis of relevant CALM MOT study data to address CALM STORY objectives. Further methodological details are articulated below.

#### 2.1.1 Theoretical Grounding

CALM STORY is theoretically guided by TVIR principles and the Lundy Model of Participation [31]. In health and mental health service contexts, a TVIC framework explicitly acknowledges the relational element of care experiences and the principles of choice, connection, collaboration, and control within care encounters [19]. TVIR extends these principles into research spaces, recognising that although research and clinical care have distinct purposes, both are nonetheless relational and thus require a relational approach. While TVIR articulates the principles that should orient research practice, it does not on its own specify how these values might be structurally operationalized with youth; this is the gap that the Lundy Model of Participation helps to address [32].

The Lundy Model of Participation is a framework for operationalizing child and youth participatory rights [31]. It is organised around four elements: space, voice, audience, and influence. The model asserts that the participatory rights of children and youth are honoured only when they are provided with the space to express their opinions, the opportunity to share their voices, an audience to hear them, and the opportunity to meaningfully influence decisions being made about and for them [31]. When considering TVIR and Lundy Model principles in parallel, a set of guiding principles emerges that centres child and youth participatory rights while considering the dynamics of interpersonal and systemic power within research relationships [32].

#### 2.1.2 Youth Engagement

Youth engagement approaches for the CALM MOT have been guided by the McCain Model of Youth‐Adult Partnerships [33] and are operationalized through an established Youth Advisory Group (YAG) [25]. The CALM MOT YAG is composed of eight youth with lived experience of, and expertise related to, MMHC. The YAG was consulted in October 2025 on CALM STORY's proposed data collection methods and interview guides; YAG feedback was integrated into the refinement of study questions and methods. Interim progress reports and consultations will continue with the YAG as CALM STORY unfolds.

### 2.2 Sampling and Recruitment

Aligned with our qualitative research paradigm, we will use purposive sampling procedures to identify and recruit approximately 30 youth to complete CALM STORY activities. Purposive sampling enables the strategic identification of participants who can offer in‐depth knowledge that is relevant to addressing the research objectives [34, 35]. Participants for CALM STORY will be recruited from the cohort of youth aged 11–19 years old enroled at the MCH‐HHS site of the CALM MOT and, through their participation in the CALM MOT, have consented to be contacted about possible participation in related studies. It is important to note that, although participants across the CALM MOT are between the ages of 11–24 years old, MCH‐HHS is a children's hospital and therefore only services youth under 18 years old. Thus, the age‐related eligibility criteria for CALM STORY for MCH‐HHS is different than that of the CALM MOT, while additionally accommodating youth who may have accessed care at MCH‐HHS at the age of 18 but turned 19 during their CALM MOT participation.

#### 2.2.1 Eligibility

##### 2.2.1.1 Youth Participants

Eligibility criteria for youth to participate in CALM STORY is directly linked to enrolment in the CALM MOT at the MCH‐HHS site. CALM STORY participants will:

i. Have signed and dated the CALM MOT informed consent form, or provided assent and have had a Substitute Decision Maker provide informed consent for the youth to participate in the CALM MOT;
ii. Be aged 11–19 years old at the time of CALM MOT screening;
iii. Be any person within age criteria who is seeking or accessing mental health services at the MCH‐HHS CALM MOT site;
iv. Be able to complete CALM MOT assessments in English.

In addition to the above, CALM STORY youth participants must:

v. Have indicated consent/assent to be contacted for additional research related to the CALM MOT;
vi. Have created a CRP as part of their baseline CALM MOT visit;
vii. Have stated a willingness to participate in CALM STORY;
viii. Have provided informed consent or assent to participate in CALM STORY;
ix. Have their own device with digital photo taking capabilities.

There are no definitive sample size requirements for inductive, longitudinal qualitative health research studies that integrate arts‐based data collection methods. Instead, methodological guidance indicates that sample size estimates should be grounded in the research aims, participant availability, as well as take into account whether there will be singular versus multiple sources of data from the same or distinct participants [34, 36, 37]. Accordingly, we ground our sampling approach within the concept of ‘information power’, weighing considerations of breadth of study aim, sample specificity, our applied theoretical grounding, anticipated quality of interview data, and analysis plan [38]. We will use administrative information from the CALM MOT to intentionally approach eligible youth across a spectrum of age and gender diversity. Given our plan to generate multiple sources of qualitative data from the same participants longitudinally, we anticipate that a total sample of approximately 30 youth between the ages of 11 to 19 for CALM STORY will be sufficient to achieve our research objectives.

### 2.3 Data Collection

#### 2.3.1 CALM Data for Secondary Analysis

##### 2.3.1.1 CALM Participant Demographic Data

With youth participant consent, core socio‐demographic data for CALM STORY participants will be extracted from the CALM MOT database for the purposes of describing our CALM STORY sample. Demographic data collected as part of participation in the CALM MOT includes self‐report information about their age, sex, gender, race and ethnicity, and education level, with information about youth's current mental health conditions generated via standardised coding of structured diagnostic interview data and/or youth self‐report of symptoms on psychometrically validated questionnaires.

##### 2.3.1.2 Audit Trail of CALM MOT Standard Operating Procedure Documentation

As part of the CALM MOT SOPs, staff team members' document examples of how and where TVIR principles have been integrated into research encounters. This documentation includes, but is not limited to: basic needs provision, relationships with research staff, safety, and experiences of distress.

#### 2.3.2 Primary Data Collection

##### 2.3.2.1 First Semi‐Structured Qualitative Interview With Doodling (CRP Specific)

As part of CALM MOT SOPs to reduce risk of emotional distress, self‐harm, and suicidal ideation or attempts, participants of the CALM MOT at MCH‐HHS collaborate with trained research staff to create an individualised Crisis Response Plan (CRP) at their baseline study visit. The CRP involves the collaborative consolidation of supportive information onto a pocket size index card that a young person can easily carry with them (e.g., in a pocket or backpack or phone holder) and easily reference during times of emotional distress [39, 40]. Additional information about CRP's can be found in the Supporting Information document (Section 7).

To address secondary objective “a” we will ask youth participants for consent to use the photocopied version of their CRP as a ‘primer’ for CALM STORY data collection. To learn about their experiences of the CRP, participants will be asked to engage in an individual qualitative semi‐structured interview that inquires about their experience of (a) collaboratively generating their CALM MOT CRP with the research team, (b) using it during research encounters, as well as (c) using it for situations, events or experiences outside of CALM MOT research visits.

During this interview, youth participants will be provided with the photocopied version of the CRP that they created at their baseline CALM MOT visit. The photocopied version of the CRP will be glued to the centre of a larger piece of blank paper, effectively creating margins for each participant to doodle in as they engage in their semi‐structured qualitative interview with research staff. Doodling, otherwise known as the act of drawing and scribbling along the margins of blank documents or text, has gained recognition as a “live method” [41, p. 3] that offers an artful outlet for knowledge that may not be easily organised or expressed verbally [42], providing a complementary contrast to the CALM MOT's highly structured, quantitative design. If CALM STORY youth choose to create a doodle, participants will be asked to verbally describe their doodles at the end of the interview.

##### 2.3.2.2 Second Semi‐Structured Qualitative Interview (CALM MOT Participation)

Five months following their first semi‐structured qualitative interview, youth participants will be asked to complete a second semi‐structured interview about their participation in the CALM MOT more broadly. Our primary objective and secondary objective “b” will be partially addressed here; the second individual qualitative semi‐structured interview for CALM STORY will inquire about the TVIR principles of connection, choice, collaboration and control, including participant perceptions of (a) the importance of consistent research staff involvement, (b) instances and experiences of relationship building and rupture with research staff, (c) opportunities to articulate their study participation preferences, and (d) other aspects of their lives they believe should be measured to capture their experiences in the CALM MOT.

Both qualitative interviews will be scheduled with the research staff member at the participant's convenience and will preferentially take place in‐person. If a participant prefers, the interview can take place virtually over Zoom. Interviews will last approximately 45–60 min. For each interview, participants will be provided with a $10 gift card of their choosing.

##### 2.3.2.3 Photovoice

To capture data related to each of our research objectives, participants will have the opportunity to participate in an eight‐part PhotoVoice workshop series with other CALM STORY participants. PhotoVoice is a participatory arts‐based method [43] that provides opportunities for those who are often pushed to the margins ‐ such as youth with mental health conditions ‐ to “represent their community and narrate their everyday experiences using their own voices” [37, p. 170]. The use of imagery and narration allows participants to share their own stories on their own terms, providing a rich visual depiction of their own lived experiences in relation to the phenomenon in question [36].

PhotoVoice is particularly well‐suited for research that is done with young people with mental health conditions [44] as a flexible, accessible, and creative format that aligns well with TVIR principles.

###### 2.3.2.3.1 Photovoice Procedures

All 30 youth who consented to participate in CALM STORY will be asked to participate in the PhotoVoice workshops, held in four groups of 7–10 youth per group. PhotoVoice participation will closely align with their 12‐month timepoint of CALM MOT participation. Each group will meet weekly, in person or over zoom, for eight sessions total. Photos will be taken on participants' own devices; workshops will be audio recorded and transcribed verbatim for analysis. For each PhotoVoice session attended, participants will receive a $10 gift card of their choosing. Participants will additionally receive a $20 gift card of their choosing for each set of weekly submitted photos.

A brief outline of the PhotoVoice process is provided in Table 1, with a more detailed description of the PhotoVoice workshops found in the Supporting Information document (Section 2).

**Table 1:** A summary of the PhotoVoice process.

**Session 1**	***Information and community‐building***
	The first session in PhotoVoice workshop series provides participants with an opportunity to get to know each other, grow comfortability with the group, gain familiarity with the PhotoVoice process, and establish some rules of engagement for the group to follow moving forward.
**Sessions 2–6**	***Photo Sharing***
	In sessions two through six, participants will engage with the facilitated PhotoVoice probing questions and share their photos with each other.
**Sessions 7–8**	***Preliminary Participatory Analysis***
	Participants will be asked to choose one photo from each week (a total of five photos per participant) to showcase in session seven. The group of participants will then partake in an adaptation of the SHOWeD analysis method (Wallerstein & Bernstein, 1988). In the final session, participants will engage in a discussion to identify some preliminary themes amongst the photos and to create a potential knowledge dissemination plan.

#### 2.3.3 Distress Response and Emotional Safety Protocol

Reflecting on personal experiences during semi‐structured interviews and PhotoVoice workshops may, for some participants, surface unexpected or intense thoughts and feelings. In keeping with TVIR, CALM STORY's design enacts the choice, connection, collaboration, and control it aims to evaluate to also respond to distress and complex emotions that come up (if at all) during research activities. The principal investigator (first author) and senior author are both trained and registered social workers with over twenty years of combined experience working with youth in community, clinical care, and research settings; research staff engaged in CALM STORY activities are required to complete training in TVIR, evidence‐based emotion coaching skills, and will be supported and closely supervised to ensure that participants are adequately supported through potential experiences of distress. The senior author, as a clinical social worker, provides ‘on‐call’ support to team members whose concern about participant distress is identified as an immediate safety concern. Additional supportive strategies include re‐affirming the voluntary nature of participation, supporting participants to use their CRP strategies during and after data collection, and providing a resource sheet of free, low‐cost, low‐barrier supports.

Though PhotoVoice was selected for its suitability for use with youth with mental health challenges, group‐based activities may not suit everyone. In these cases, participants may choose an adapted individual format, completing all activities independently with the support of a dedicated research staff member: taking photos on their own and completing individual interviews (recorded and transcribed verbatim) that mirror the format and questions of group workshops.

To protect confidentiality, PhotoVoice Session 1 includes orientation to research ethics and guidance on appropriate photo content, covering privacy (e.g., avoiding others' faces) and confidentiality (e.g., avoiding other identifying details like school names or home addresses). Photos that may jeopardise privacy and confidentiality will have those aspects of the photo blurred or blocked out using editing software.

## 3 Data Analysis and Rigour

Rather than functioning as discrete data sources, the two interviews, doodling activity, and PhotoVoice Workshops serve as complementary entry points into youth's TVIR perceptions and experiences. These sources of data will therefore be triangulated using framework‐based thematic analysis to build a layered, contextualised understanding of the perceived value and impact of TVIR approaches to support longitudinal research participation [45]. Data and associated field notes will be managed in NVivo. Two members of the research team who are trained in qualitative research and data analysis methods, will independently review and familiarise themselves with all data in full. Subsequently, an a priori analysis matrix that operationalizes TVIR's principles of choice, connection, collaboration, and control alongside the Lundy Model of Participation's elements of space, voice, audience, and influence [45] will function as pre‐defined codes for the first level of data analysis (see Supporting Information File, Section 3). These codes will be applied to all sources of data – a process referred to as indexing – resulting in the development of a framework matrix. Where deemed relevant and appropriate to addressing the research objective, the data analysts will integrate audit trail information into these procedures. Subsequnetly, the research team members will iteratively review the framework matrix to synthesise all data using inductively generated themes that are attuned to participant percpetions and experiences broadly, as well as whether those experiences and perceptions shift over time (if at all). The themes will reflect patterns and variation in how youth perceive and experience TVIR and its specific strategies within the CALM MOT, illuminating both the individual and intersecting TVIR and Lundy Model principles within youth's accounts of participation in longitudinal mental health research.

So as not to incorrectly assume or attribute meaning to visual materials by those who did not create them, photographs (CRP doodles and PhotoVoice images) will not undergo visual analysis by the research team. Instead, participants' accompanying narratives captured in interview and workshop transcripts will be included in the analysis. With consent, de‐identified quotations and photographs may be used in knowledge dissemination, with identifying features removed or obscured as appropriate.

Demographic data extracted from the CALM MOT [25] will descriptively characterise the CALM STORY sample. Rather than inferential statistics, these data will contextualise the qualitative findings and support interpretation of themes in relation to participants' age and gender.

Trustworthiness will be supported through strategies addressing credibility, transferability, dependability, and confirmability [46]. Credibility will be strengthened through prolonged engagement, triangulation across interviews, PhotoVoice Workshops, and integration of thematic outputs from the participatory analysis workshop into team‐based thematic interpretation. Transferability will be supported through rich description of study context, participants, processes, and youth experiences, enabling readers to assess relevance to other youth mental health research settings. Dependability and confirmability will be addressed through a maintained study audit trail, reflexive documentation, and transparent analytic decision‐making.

Importantly, the research team is dedicated to an ongoing practice of critical reflexivity which encourages continued consideration of how “values, assumptions, biases, and actions have been informed by larger systems of power and how they help to uphold, reproduce, and reconstitute oppression and privilege” within the research process [47, p. 3]. This is particularly relevant for TVIR in recognising the interpersonal and systemic power dynamics that may exist between researchers and participants, and which may influence data collection and analysis. In the spirit of critical reflexivity within the research process, both the first and senior author have offered reflexivity statements in the Supporting Information file (Section 4).

## 4 Ethical Considerations

CALM STORY was reviewed by the Hamilton Integrated Research Ethics Board (HiREB; Project ID #: 18993) and approved on April 17, 2026. At the time of protocol submission to the journal, CALM STORY data collection had not been initiated.

In addition to adhering to procedural ethics principles of respect for persons, concern for welfare, and justice [48] the research team will critically consider “ethics in practice” [49, p. 262]. By this, it is meant that research staff will reflexively analyse how ethical conduct exists within each research encounter, including conversations about relational and power dynamics that sometimes exist between youth and adults, researchers and participants, and participants with each other. Consistent with TVIR principles, consent will be treated as an ongoing moment‐to‐moment process, and research staff will be attentive to participant discomfort, distress, and autonomy throughout data collection. We are committed to maintaining high ethical standards, both procedurally and in practice, and will engage in continuous team‐based reflection to support ethical, respectful, and youth‐centred research conduct.

## 5 Discussion

CALM STORY addresses a critical gap in longitudinal youth mental health research: the lack of empirical, youth‐centered evidence on how TVIR principles are perceived and experienced within the research process itself. While studies like the CALM MOT generate important data on mental health trajectories, explanatory mechanisms, and outcomes, far less is known about how the relational strategies of choice, connection, collaboration and control are valued and experienced by youth over time, nor whether youth are afforded genuine space, voice, audience, and influence within research participation. By centreing youth participation experiences in a large scale, quantitative longitudinal study, CALM STORY shifts the focus from youth as data sources to youth as knowledge and rights holders, generating evidence to inform how research is designed and implemented.

### 5.1 Anticipated Limitations and Study Strengths

CALM STORY has several anticipated limitations. First, there may be self‐selection bias among CALM MOT participants willing to participate in further research, or in arts‐based research activities. This includes a potential bias towards those who have the time and resources to participate in sustained, weekly activities such as the PhotoVoice workshops. While the study design includes flexible participation options (e.g., individual PhotoVoice) and compensation for time, these biases cannot be assumed to be fully mitigated.

A further limitation is that CALM STORY is a single‐site study, which may limit transferability. Integration of TVIR principles into site SOPs at MCH‐HHS was made possible because of the expertise of the site principal investigator (senior author); implementing similar procedures and staff training elsewhere would require comparable interest, buy‐in, and resources. This limitation may extend to fee‐for‐service contexts: as the CALM MOT operates across six publicly‐funded specialty mental health care sites across Canada, findings may not generalise to services requiring private payment or insurance. Finally, CALM STORY is being conducted in English, which may limit transferability of findings to linguistically diverse populations.

Despite these limitations, CALM STORY has many strengths. Embedded within a longitudinal cohort study with multiple data points, it offers youth ample opportunity to engage with and reflect upon their participation experiences over time; given the unique nature of the CALM MOT, this will yield novel insight into sustained research participation among youth with complex mental health needs.

CALM STORY's use of an accessible, participatory arts‐based methodology is a further strength. Arts‐based methodologies offer children and youth with varied knowledges and communication preferences opportunities to engage in research processes, meaningfully including perspectives that might otherwise be excluded [50]. This approach aligns with a participatory rights framework, exemplified by the Lundy Model of Participation, further reinforcing CALM STORY's TVIR and rights‐based approach to youth participation.

### 5.2 Knowledge Dissemination

Knowledge created through CALM STORY will be communicated in several ways and shared with a broad range of audiences, with dissemination strategies identified and implemented in collaboration with the CALM MOT YAG. To date, we plan to share study findings via: a research policy brief that outlines recommendations for youth mental health research, a peer reviewed publication, and conference presentations. The knowledge generated in CALM STORY will additionally be used to inform the refinement and expansion of TVIR training embedded in the CALM MOT, as well as broader training opportunities for the youth mental health research field. The latter includes TVIR‐focused workshops through the VEGA Project [19], a pan‐Canadian initiative led by the senior author at McMaster University and which is advancing trauma‐ and violence‐informed health sector responses in practice and research.

### 5.3 Potential Impact and Recommendations

Given the limited empirical literature on the integrating trauma‐ and violence‐informed principles in research contexts, CALM STORY will provide timely, youth‐centred insight into how TVIR may be embedded within longitudinal, quantitative research designs. While we cannot offer evidence‐based recommendations for TVIR prior to CALM STORY's completion, we suggest that longitudinal cohort and clinical trial designers may benefit from preliminary, literature‐informed considerations in the interim (e.g., Bernier & Kimber, Under Review; Isobel, 2021; Kimber & MacMillan, In Press) as well as the broader youth engagement literature that speaks to the nuances of working safely with young people at risk of or who have experienced trauma (e.g., [51, 52]). CALM STORY's own evidence‐based recommendations will follow study completion.

## 6 Conclusion

This study protocol outlines the steps for a qualitative, multimethod, arts‐based research study which aims to better understand the experiences and perspectives of longitudinal quantitative research participation among youth. The detailed description of study rationale, data collection methods, and data analysis is intended to support researchers who are interested in engaging in similar work. Notably, the protocol also attends to important ethical considerations that extend beyond procedural ethics, including the application of TVIR principles within longitudinal research paradigms. Publishing this protocol before study implementation offers guidance for other researchers who endeavour to pursue participatory, arts‐based research about youth experiences.

Understanding the perceived value, impact, and challenges of TVIR approaches has important implications for ethical research practice beyond procedural ethics, participant safety, and retention, particularly for youth who are at greater risk of having their lived experience underrepresented in the evidence base. By generating youth‐informed insights into how TVIR strategies are operationalized and experienced, this study will support the development of more relational and sustainable research environments, thereby strengthening the quality and integrity of future longitudinal youth mental health research.

## Author Contributions

**Alexe Bernier:** conceptualisation, writing – original draft, methodology, writing – review and editing, project administration. **Zita de Snoo:** writing – original draft, writing – review and editing, methodology. **Nawal Masood:** writing – original draft, writing – review and editing, methodology. **Jawahir Al Bayati:** writing – review and editing. **Louise Gallagher:** methodology, writing – review and editing. **Melissa Kimber:** conceptualisation, funding acquisition, writing – review and editing, methodology, supervision.

## Ethics Statement

This study received ethics approval from the Hamilton Integrated Research Ethics Board (HiREB Project ID: 18‐993).

## Conflicts of Interest

The authors have no conflicts of interest.

## Supporting information


Supporting File

## Data Availability

The data collected for this study will not be made publicly available for use beyond the findings generated for this project.
