# Supplementary material for: Exploring the Integration of Trauma‐ and Violence‐Informed Research Principles in Longitudinal Youth Mental Health Research: An Arts‐Based Protocol
Source: Health Expect. 2026 Oct 29;29(6):e70941. doi: 10.1111/hex.70941 (PMC13717300; doi:10.1111/hex.70941)
Supplement: Supplementary file 1 [file HEX-29-e70941-s001.docx]

**CALM STORY**

**Section 1: Crisis Response Plan (Contextual Information)**

Originally developed as a tool to use with military veterans experiencing suicidal ideation and attempts,^1^ the CRP has additionally been used elsewhere to support general adult populations with histories of suicidality, as well as with youth at high risk of suicide.^1,2^ While CRP’s do share many characteristics with traditional safety plans, CRP’s are notably different in that they are rooted in and guided by participants’ own lived experiences, asking that they draw from previous experiences of crisis and distress.^2^ The collaborative creation of a CRP includes “identifying one's personal warning signs, using coping strategies, activating social support, and accessing professional services”.^1(p65)^ An example of a typical CRP is depicted in Figure 1.

Figure 1. An example of a Crisis Response Plan as created by a member of the research team.

The intention of creating the CRP with youth participants at their baseline visit is to ensure that participants have made explicit the tools and strategies that they find helpful for managing and reducing emotional distress that may arise within or between CALM MOT research visits. As part of CALM MOT Standardized Operating Procedures for risk management and distress response, and upon collaboratively generating their CRP, research staff request verbal permission from the CALM MOT participant to keep a photocopied and de-identified version of the participant’s CRP (labelled only with their unique CALM MOT study identifier) in a binder in a locked cabinet in the research visit office.

**Section 2: PhotoVoice**

As described by Wang and Burris^3^, the creators of PhotoVoice methodology, key components of PhotoVoice should include (1) an information and community-building session, (2) regular group sessions, and (3) opportunities for participatory analysis. Importantly, the methodological guidelines for PhotoVoice indicate the need to provide a general structure and conceptual focus for each of the PhotoVoice workshop sessions. However, as is customary in participatory qualitative approaches, the conceptual focus of sessions can be amended as sessions and groups unfold to ensure robust exploration of concepts and themes relevant to the research participants *and* addressing the study’s research question(s).

***PhotoVoice Procedures***

Each PhotoVoice workshop will be co-facilitated by two trained research staff. If a participant chooses to engage in an adapted PhotoVoice process as an individual (instead of participating in group workshops), all sessions will follow the same structure as that intended for the group workshop but will instead involve individual weekly interviews with a research staff.

**Week 1: Information and Community-Building**

The first week in PhotoVoice is designed to provide participants with an opportunity to get to know each other, grow comfortability with the group, and to gain familiarity with the PhotoVoice process. In brief, session 1 will adhere to the following structure:

- Confidentiality and Group Guidelines
- Introductions
- Ice-breaker activity
- PhotoVoice 101 presentation
- Information about how to take a photo for PhotoVoice
- Ethical considerations and expectations
- Plans for future sessions

To describe this process in further detail, participants will gather together either in-person or virtually over Zoom with the co-facilitators to meet each other. The session will begin withintroductions of the co-facilitators. The co-facilitators will then lead a discussion about group privacy and confidentiality and generate ground rules / guidelines for participation with the participants.

Following the discussion about confidentiality, participants will be asked to introduce themselves using their chosen pseudonyms and the co-facilitators will guide the group through an ice-breaker activity.

Next, an overview of PhotoVoice will be provided by the co-facilitators. The co-facilitators will lead an education session as part of an informal, conversational style group discussion. Through this discussion participants will learn about PhotoVoice and will receive information about how to take photos for their participation.

Participants will also be instructed regarding the ethical considerations of taking photographs. For example, participants will be encouraged not to identify themselves in the photos and instead include only a hand but no full bodies or faces. Or, if participants choose to include their face in a photo a conversation will be had about the ethical implications of this and the use of this photo in dissemination activities. It will also be discussed with participants to not include photos or identifiable information of other people who are not involved in the research. If they include photos of other people, the research team will work with them to blur out or remove the person from the photo.

Following this portion of the session, participants will review the study research questions, the goals of the weekly sessions, and create a plan for photo collection between Weeks 1 and 2.

**Weeks 2-6: Generating Photos that Respond to the Research Questions**

In sessions two to six, participants will take, collect, and share photos with the co-facilitators that respond to the research questions outlined below. Participants will be encouraged to take as many photos as they would like in response to the questions outlined below.

During the group sessions, the photos will be reviewed and discussed as a group. Over these five weeks, participants will answer the following questions through the photos they generate and the stories and experiences they share during the weekly audio-recorded sessions.

Week 1 (Experiences of General Participation):

- What has it been like for you to participate in the CALM MOT?
- What have you enjoyed or disliked about participating in the CALM MOT?

Week 2: (Experiences of Participating Locally)

*Context: as you all know, McMaster Children’s Hospital is one site out of six that is doing the CALM MOT. At our site, we have tried our best to create a research environment that feels supportive, welcoming, and inclusive. To do this, we took a trauma- and violence-informed approach to doing research. Key principles of a trauma- and violence-informed approach to research are choice, connection, collaboration, and control.*

*We know that young people experience things like poverty sexism, racism, ableism, and more – and we know that this impacts youth mental health, your experiences with mental health care, and your experiences with research. It was really important to us that we acknowledged that and named it as it is. It was important to us that participants feel like they have choices in how they participate, that they are able to collaborate with research staff, and that they are able to voice their thoughts and opinions about the study when they have them.*

***if relevant to the group**: some examples of this that we tried to implement are the choices in gift cards and snacks that we provided, the way that we made the crisis response plan with all of you, asking if you wanted your caregivers involved in the study with you, providing you with engagement gifts and sending you birthday cards, and the way the visit room was designed.*

- How did you experience choice, connection, collaboration, and control in the CALM MOT?
- How could the CALM MOT do a better job of giving participants choice, connection, collaboration, and control?
- How else did you experience a trauma- and violence-informed approach in your CALM MOT participation experience?

Week 3 (Experiences of Participating Locally):

*Context: remind participants of conversation from week before about TVIR.*

- How have your interactions with research staff shaped your experiences in the CALM MOT?
  - *Contextual prompt: the entire research staff team identified as women. The majority of the staff team was white. Did things like this shape your experiences?*
- What has your experience with the study space (downtown Hamilton, study visit room, or virtual) been like?
- What was it like to come to in-person study visits?
  - *Contextual prompt: some of you bring yourselves or are brought to study visits by car by their parents or siblings. Others were brought to study visits by taxicab, uber, or by the city bus. What has that been like for you?*

Week 4 (Perspectives on Youth Mental Health Research):

- What do you think researchers should consider when doing research with youth with mental health conditions?
- How do you see the connection between mental health research and clinical mental health care? How do you see them as separate?

Week 5 (Hopes for the Future of Youth Mental Health Research):

- How do you think we can encourage young people to participate in mental health research in the years to come? What is important for researchers to consider?
- Why do you think it’s important to center young people in youth mental health research?
- What do you hope other young people will get out of your participation in the CALM MOT?

**Week Seven: Preliminary Participatory Analysis**

In advance of week 7, participants will be asked to choose one photo from each week (five photos per participant) to showcase. The group of participants will then partake in the SHOWeD analysis method^4^ together to identify some points of significance related to the photos that they took.

The photos chosen by participants for each week will be shown together. Participants will be asked to engage with the following questions as per an adaptation of the SHOWeD analysis method^5^ to encourage conversation amongst participants.

- SHOWeD analysis method
  - What do you *See* in the picture?
  - What is *Happening* in the picture?
  - How is it related to *Our* research experiences with youth mental health research?
  - *Why* does this matter?
  - How can we be *Empowered* by understanding what research is like for young people?
  - What can we *Do* to improve youth experiences of mental health research?

The goal of the SHOWeD analysis method in this session is to start identifying potential patterns in the photos in relation to the research question.

**Week 8: Preliminary Identification of Themes and Series Closure**

In the final session, participants will engage in a discussion to identify some preliminary themes amongst the photos. The co-facilitators will confirm and document the preliminary themes that the participants have identified through conversation.

The co-facilitators will inquire if any of the participants are interested in being further involved in data analysis and/or knowledge dissemination. Participants will be thanked for all of their contributions to the workshop series and provided with information about next steps in the research.

**Section 3: Analysis Matrix**

|  | **TVIR** | | **Lundy Model** | |
| --- | --- | --- | --- | --- |
| **Participant A** | **Interview #1** | | | |
|  | Choice |  | Space |  |
|  | Collaboration |  | Voice |  |
|  | Connection |  | Audience |  |
|  | Control |  | Influence |  |
|  | **Interview #2** | | | |
|  | Choice |  | Space |  |
|  | Collaboration |  | Voice |  |
|  | Connection |  | Audience |  |
|  | Control |  | Influence |  |
|  | Notes: | | | |
|  | **TVIR** | | **Lundy Model** | |
| **Participant B** | **Interview #1** | | | |
|  | Choice |  | Space |  |
|  | Collaboration |  | Voice |  |
|  | Connection |  | Audience |  |
|  | Control |  | Influence |  |
|  | **Interview #2** | | | |
|  | Choice |  | Space |  |
|  | Collaboration |  | Voice |  |
|  | Connection |  | Audience |  |
|  | Control |  | Influence |  |
|  | Notes: | | | |
|  | **TVIR** | | **Lundy Model** | |
| **PhotoVoice Session 2** | Choice |  | Space |  |
|  | Collaboration |  | Voice |  |
|  | Connection |  | Audience |  |
|  | Control |  | Influence |  |
|  | Notes: | | | |
|  | **TVIR** | | **Lundy Model** | |
| **PhotoVoice Session 3** | Choice |  | Space |  |
|  | Collaboration |  | Voice |  |
|  | Connection |  | Audience |  |
|  | Control |  | Influence |  |
|  | Notes: | | | |

**Section 4: Reflexivity Statements**

[Principal Investigator/First Author - Name Removed for Peer Review]

The first author holds a PhD in social work, with extensive training in both qualitative and arts-based research methodologies. She is a white, cisgender, heterosexual early-career researcher whose research is informed by over 8 years of community-based social work practice working with women and girls in violence prevention, community development, and activist contexts. With a research program centered around honoring child and youth participatory rights, she is now bridged between the social and health sciences where she works to center children and youth’s perspectives in research that is done with, for, and about them. This commitment is carried forth via her critical orientation, and as such, she is particularly attuned to and interested in issues of adultism and child-adult power imbalances within researcher-participant relationships.

[Senior Author - Name Removed for Peer Review]

The senior author is a Registered Social Worker and health services researcher whose clinical and research career has centred on intimate partner violence, child maltreatment, and youth mental health. Her perspective is shaped by more than 10 years of frontline clinical practice providing mental health assessment and intervention to youth alongside maintaining her research program, which positions her as both a clinician embedded in the systems she studies and a researcher studying those systems from within. She has a PhD in health research methodology, with extensive training in quantitative, qualitative and mixed method research paradigms. Her work is grounded in trauma- and violence-informed principles and a commitment to youth's participatory rights, which shapes how she approaches this research: as a collaborative process centering the perspectives of young people. As a mid-career, cisgender, white, heterosexual woman with lived experiences of violence and mental health challenges in her youth, this has shaped her motivation to engage in research and practice, thoughtfully and safely.

**References**

1.	Bryan CJ, Mintz J, Clemans TA, et al. Effect of crisis response planning vs. contracts for safety on suicide risk in U.S. Army Soldiers: A randomized clinical trial. *J Affect Disord*. 2017;212:64-72. doi:10.1016/j.jad.2017.01.028

2.	Gorka SM, Fuller A, Toleson S, et al. Feasibility Trial of a Single Session of Crisis Response Planning for Youth at High Risk for Suicide. *Behav Ther*. Published online December 2025:S0005789425001285. doi:10.1016/j.beth.2025.12.005

3.	Wang C, Burris MA. Photovoice: Concept, Methodology, and Use for Participatory Needs Assessment. *Health Educ Behav*. 1997;24(3):369-387. doi:10.1177/109019819702400309

4.	Wallerstein N, Bernstein E. Empowerment Education: Freire’s Ideas Adapted to Health Education. *Health Educ Q*. 1988;15(4):379-394. doi:10.1177/109019818801500402

5.	Wang CC. Photovoice: A Participatory Action Research Strategy Applied to Women’s Health. *J Womens Health*. 1999;8(2):185-192. doi:10.1089/jwh.1999.8.185
